# Enriching Surface‐Accessible CO_2_ in the Zero‐Gap Anion‐Exchange‐Membrane‐Based CO_2_ Electrolyzer

**DOI:** 10.1002/anie.202214383

**Published:** 2022-12-13

**Authors:** Qiucheng Xu, Aoni Xu, Sahil Garg, Asger B. Moss, Ib Chorkendorff, Thomas Bligaard, Brian Seger

**Affiliations:** ^1^ Surface Physics and Catalysis (Surf Cat) Section Department of Physics Technical University of Denmark 2800 Kongens, Lyngby Denmark; ^2^ CatTheory Center Department of Physics Technical University of Denmark 2800 Kongens, Lyngby Denmark; ^3^ Department of Energy Conversion and Storage Technical University of Denmark 2800 Kongens, Lyngby Denmark

**Keywords:** Carbon Dioxide Reduction, Electrocatalysis, Membrane-Electrode Assemblies, Pulse Methods

## Abstract

Zero‐gap anion exchange membrane (AEM)‐based CO_2_ electrolysis is a promising technology for CO production, however, their performance at elevated current densities still suffers from the low local CO_2_ concentration due to heavy CO_2_ neutralization. Herein, via modulating the CO_2_ feed mode and quantitative analyzing CO_2_ utilization with the aid of mass transport modeling, we develop a descriptor denoted as the surface‐accessible CO_2_ concentration ([CO_2_]^SA^), which enables us to indicate the transient state of the local [CO_2_]/[OH^−^] ratio and helps define the limits of CO_2_‐to‐CO conversion. To enrich the [CO_2_]^SA^, we developed three general strategies: (1) increasing catalyst layer thickness, (2) elevating CO_2_ pressure, and (3) applying a pulsed electrochemical (PE) method. Notably, an optimized PE method allows to keep the [CO_2_]^SA^ at a high level by utilizing the dynamic balance period of CO_2_ neutralization. A maximum *j*_CO_ of 368±28 mA cm_geo_^−2^ was achieved using a commercial silver catalyst.

## Graphical abstract

A descriptor called the surface‐accessible CO_2_ concentration ([CO_2_]^SA^) is developed, which enables to indicate the transient state of the local [CO_2_]/[OH^−^] ratio and helps define the limits of CO_2_‐to‐CO conversion. Three general tactics are developed to enrich [CO_2_]^SA^ for high current electrolysis: (1) increasing the catalyst layer thickness, (2) elevating CO_2_ pressure, and (3) employing the pulse electrochemical method.

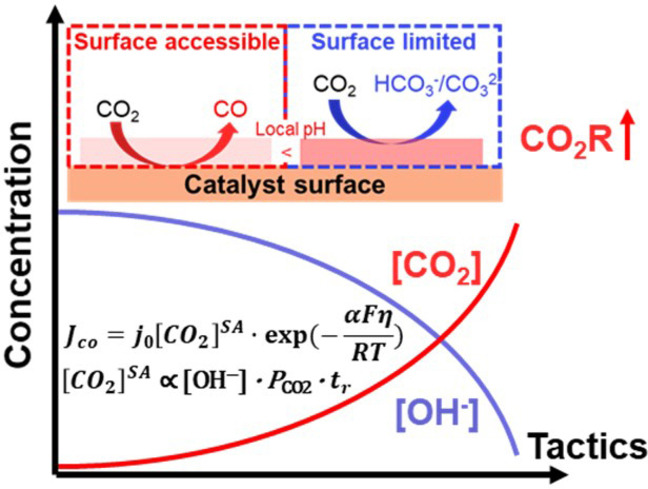

## Introduction

**Figure 1:**
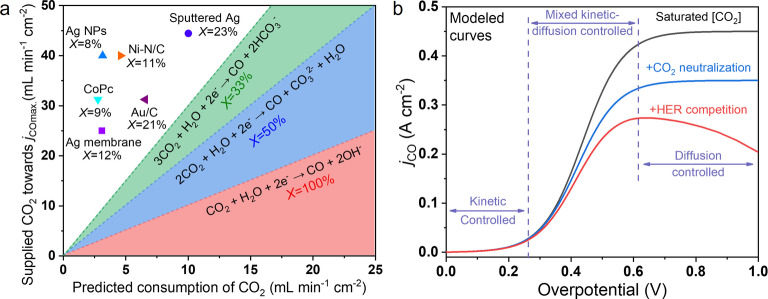
a) Statistical analysis of CO_2_ utilization in the AEM‐based zero gap CO_2_ electrolyzer for CO_2_‐to‐CO conversion (room temperature, 100 % *P*_CO2_).[^6^, ^7^] b) Kinetic analysis of linear‐polarization curves based on the assumed electrochemical CO_2_‐to‐CO conversion processes.

CO_2_ electrolysis to produce value‐added fuels or chemicals has emerged as one of the most promising technologies for a sustainable and carbon‐neutral future.^[1]^ Among various electrolysis products, CO, with a large market demand, is the closest to commercialization.^[2]^ Many recent studies have successfully produced CO at industrial‐level current densities (*j*>200 mA cm^−2^) by utilizing gas‐diffusion‐electrodes (GDE) akin to the fuel cell and electrolysis communities.^[3]^ In these cases, the GDE provides a significantly thinner diffusion boundary layer that helps to overcome the CO_2_ mass transfer limitations present in aqueous systems.^[4]^ Moreover, the anion‐exchange membrane (AEM) based zero‐gap electrolyzer approach has attracted much attention due to the advantages of offering an alkaline interface environment that impedes the competing hydrogen evolution reaction (HER) and which possesses less ohmic resistance loss compared to a flow‐cell system.^[5]^ However, the CO_2_ utilization in such AEM‐based systems has substantial room for improvement due to CO_2_ neutralization, during which the CO_2_ feed reacts with in situ generated OH^−^ forming CO_3_^2−^/HCO_3_^−^ at the cathode side. These species crossover to the anode side leading to a ≈50 % loss of CO_2_.^[6]^ As an example, Figure 1a exhibits a statistical analysis of CO_2_ utilization of recently reported zero‐gap AEM‐based electrolyzer studies for CO_2_‐to‐CO conversion.[^6^, ^7^] In detail, a much higher CO_2_ inlet feed was required to achieve the maximum partial current density of CO (*j*_CO_) when compared to the predicted CO_2_ consumption including neutralization, showing a low CO_2_ conversion rate (*X*).[^6^, ^7^] Such low conversion rates reveals greatly enhanced mass transfer issues that occur in the micro‐environments near the catalyst at large current densities. These low conversion rates also will increase downstream separation costs thereby reducing the economic feasibility.

Along with changes in operating conditions, CO_2_ neutralization provides diverse micro‐environments near the catalyst surface.[^7a^, ^8^] A few modeling studies have reported that the buffering function of the CO_2_ neutralization process stabilizes the local pH at the expense of reducing the local [CO_2_].^[9]^ In general, an intertwined reaction mechanism involving the kinetics and mass transfer of both CO_2_R and HER needs to be considered. Three modelled linear‐polarization curves of CO_2_‐to‐CO reaction at different assumed conditions are displayed in Figure 1b (detailed parameters used for this model are described in the Supporting Information). This figure shows the *j*_CO_ at three marked overpotential ranges are controlled by the intrinsic catalyst kinetics, the mixed kinetic‐diffusion, and CO_2_ mass‐transfer diffusion, respectively. A high local [CO_2_] favors both an increase in the reaction rate and the limiting *j*_CO_. In ideal conditions, the maximum limiting *j*_CO_ (black curve) will be achieved when the [CO_2_] is saturated near the catalyst surface, which is ≈34 mM at room temperature in an aqueous electrolyte. However, when CO_2_ neutralization occurs, the local [CO_2_] decreases and reaches a new equilibrium, causing a lower limiting *j*_CO_ (blue curve). By further considering the competing HER that will reduce FE_CO_ with an increasing overpotential, the *j*_CO_ will show a volcano type curve behavior and a lower limiting value at a higher overpotential. This volcano effect has actually been seen in the work by Larrazabal et al. where the *j*_CO_ is maximal at 200 mA cm^−2^ (at *j*_total_=250 mA cm^−2^) and then decreases at higher total current densities.^[6]^ The above analysis reveals the CO_2_ neutralization process severely obstructs CO_2_ mass transfer and is one of the major constraints for better CO_2_ utilization. Although substantial quantitative analyses have been reported in the ocean acidification field regarding CO_2_ neutralization,^[10]^ little of this knowledge has been transferred to spur the development of CO_2_ electrolysis. To the best of our knowledge, there is still a lack of investigations related to the neutralization chemistry of local CO_2_ and OH^−^ concentrations at large‐current density conditions and concomitantly developed strategies to detour such internal limitations.

Herein, using a commercial silver catalyst, we systematically explored the effect of changing the CO_2_ feed mode (e.g., flow rate, partial pressure, *etc*.) in a zero‐gap AEM‐based electrolyzer on CO_2_ neutralization and the micro‐environment. By combining a series of quantitatively analyzed experimental results with theoretical calculations, we found that surface accessible CO_2_ concentrations ([CO_2_]^SA^), which is a function of the local [CO_2_]/[OH^−^] ratio, is a good descriptor for CO_2_R performance at high current densities where mass transfer issues often dominate.

To enrich the [CO_2_]^SA^ in the vicinity of the catalyst, we developed three general strategies: (1) increasing catalyst layer thickness, (2) elevating CO_2_ pressure, and (3) applying a pulsed electrochemical method. Consequently, a *j*_CO_ of 368±28 mA cm^−2^ was achieved through an optimized pulsed‐electrochemical method. Under these conditions, the cell can maintain a FE_CO_ of >70 % for 13 h at 500 mA cm^−2^.

## Results and Discussion

**Figure 2:**
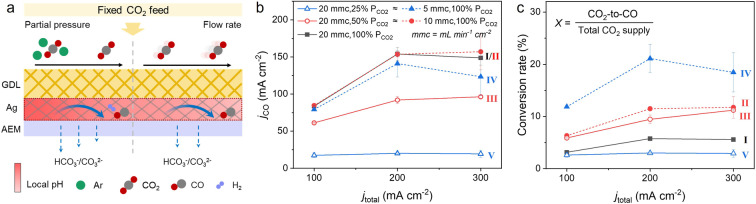
a) Schematic illustration of changing CO_2_ feed mode affecting the micro‐environment in the membrane electrode assembly (MEA). b) Partial current density of CO (*j*_CO_) and c) CO_2_‐to‐CO conversion rate at different CO_2_ feed modes and operating conditions. The unit of mL min^−1^ cm^−2^ (mmc) represents the gas flow volume per minute per active electrode area.

To explore how CO_2_ neutralization affects the local CO_2_ and OH^−^ concentration, experiments were performed by controlling the partial pressure (*P*_CO2_), flow rate (*υ*_CO2_) and applied current densities (*j*_total_), as illustrated in Figure 2a. The *P*_CO2_ will regulate the extent of CO_2_ neutralization in the catalyst vicinity and the CO_2_ coverage on the catalyst surface; the *υ*_CO2_, as a dependent parameter, will show a combined effect of the physical transport behavior of CO_2_ and the *P*_CO2_, depending on CO_2_ supply and consumption; the *j*_total_ will affect the local OH^−^ concentration due to the in situ CO_3_^2−^/HCO_3_^−^ formation process. In the experiments, we implemented five different CO_2_ feed conditions (Table 1) at *j*_total_ ranging from 100 to 300 mA cm^−2^. They can be further classified into three groups according to the total CO_2_ supplied. For example, by matching the flow rate and partial pressure, **II** (100 % P_CO2_ and 10 mL min^−1^ cm^−2^) and **III** (50 % P_CO2_ and 20 mL min^−1^ cm^−2^) conditions have the same total CO_2_ supply of ≈7×10^−6^ mol s^−1^. A silver membrane (Ag‐M, purity >99.97 %) with a hydrophilic surface was chosen as the baseline cathode catalyst since it is commercial (i.e., reproducible) and has uniform pore sizes (Figure S1). These features allow both gas and ions to easily access or leave the catalyst surface.

**Table 1:** CO_2_ feed mode and resulting total CO_2_ supply amount in the experiments.

	**Experimental conditions** ^[a]^		**Total CO_2_ supply**
1	**I**: 100 % P_CO2_, 20 mL min^−1^ cm^−2^,		≈14×10^−6^ mol s^−1^
2	**II**: 100 % P_CO2_, 10 mL min^−1^ cm^−2^	**III**: 50 % P_CO2_, 20 mL min^−1^ cm^−2^	≈7×10^−6^ mol s^−1^
3	**IV**: 100 % P_CO2_, 5 mL min^−1^ cm^−2^	**V**: 25 % P_CO2_, 20 mL min^−1^ cm^−2^	≈3.5×10^−6^ mol s^−1^

[a] The flow rate may have a deviation of ±0.1 mL min^−1^ cm^−2^ due to the resolution of mass flow controllers; humidified CO_2_ is used in all experiments.

Figure 2b shows the electrochemical CO_2_ to CO performance (*j*_CO_) at different operating conditions. Detailed information is provided in Figure S2 and S3 including cell voltage and faradic efficiency (FE). Figure 2b shows that decreasing the *P*_CO2_ dramatically drops the *j*_CO_. As it is known that the rate limiting step of CO_2_ to CO is the first electron transfer,^[11]^ it would reason that the activity would scale linearly with CO_2_ concentration. At beyond 200 mA cm^−2^, it appears the *j*_CO_ has been reached for this experimental design, and the *j*_CO_ of **III** is approximately half that of **II** as one would expect.

As mentioned above, operating with a low *υ*_CO2_ shows a complex effect of mass transfer and *P*_CO2_. The in situ formed gas products would dilute the reactant gas and thus decrease the *P*_CO2_. To verify this, the *υ*_CO2_ was reduced from 20 to 5 mL min^−1^ cm^−2^. Figure 2b shows that *j*_CO_ was not affected notably when *j*_total_=100 mA cm^−2^. Combining this with the CO_2_‐to‐CO conversion rate (*X*) in Figure 2c, where *X* proportionally increases with an increase of *υ*_CO2_ (i.e., from 3.1 % with **I** to 6.2 % with **II** or 11.9 % with **IV**, ≈2 or 4 times), this suggests that the mass transfer and the *P*_CO2_ are not limited in these conditions. Interestingly, further increasing *j*_total_ would amplify the effect of reduced *P*_CO2_ due to an increased consumption of CO_2_ as well as an increased formation of CO and H_2_. By using the FE of gas products at different *υ*_CO2_, the CO_2_ partial pressures at the cathodic outlet (*P*_CO2‐outlet_) were estimated (Figure S4). The details are provided in Supporting Information. This shows the *P*_CO2‐outlet_ drops from ≈90 % to ≈70 % when the *υ*_CO2_ decreases from 20 to 5 mL min^−1^ cm^−2^ at *j*_total_ of 300 mA cm^−2^. This decrease in partial pressure leading to a decrease in limiting *j_CO_* agrees with the trend seen between **I** and **IV**. A comparison of **IV** and **V** with each having the same amount of CO_2_ supply also clearly shows that *P*_CO2_ seems to be the dominating factor on the *j*_CO_ rather than the *υ*_CO2_, and this tendency is exacerbated with increasing *j*_total_. These results highlight that both *P*_CO2_ and *j*_total_ control the micro‐environment around the catalyst, thus their quantitative analyses of CO_2_ utilization were elaborated as follows.

**Figure 3:**
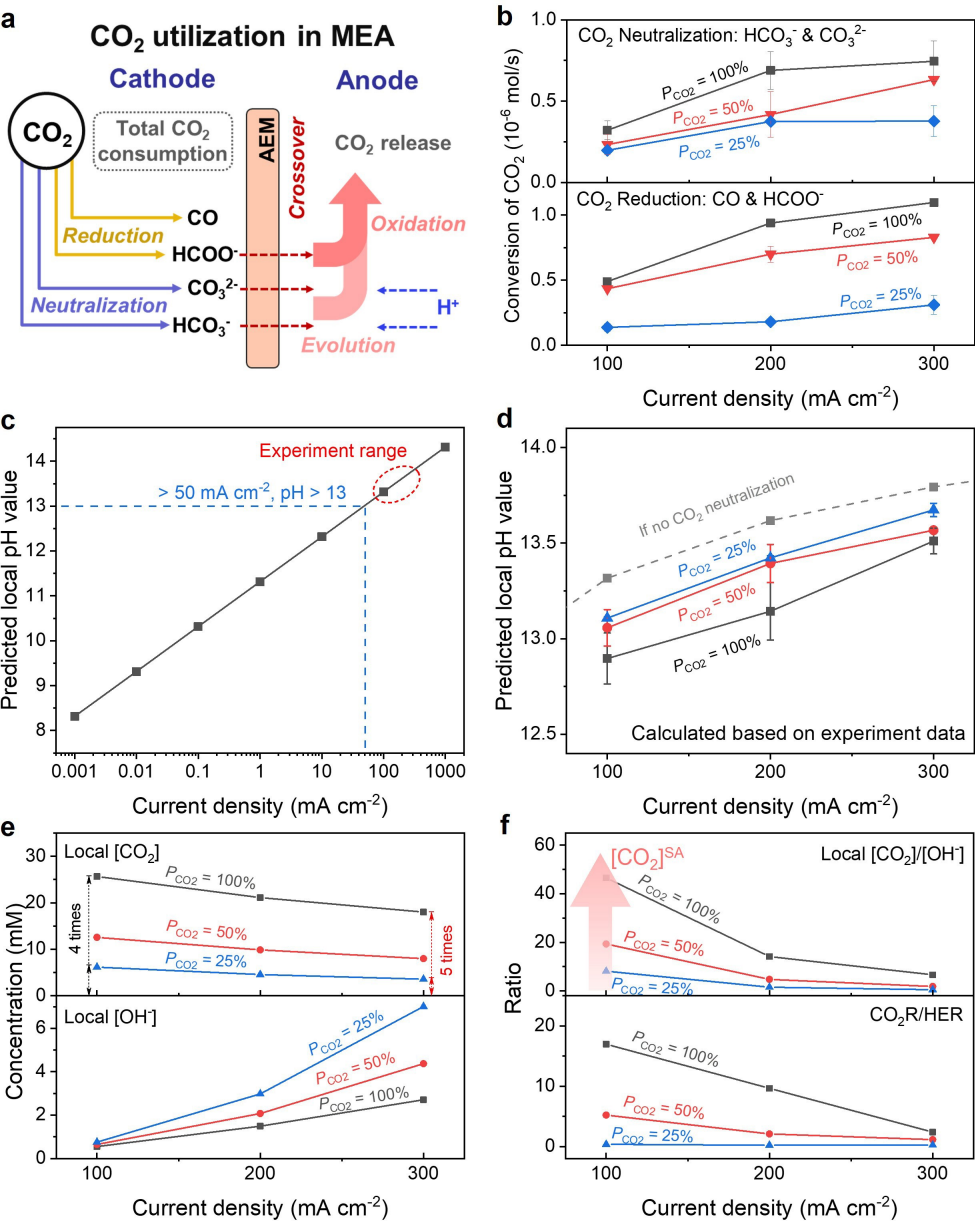
Quantitative and theoretical analyses of CO_2_ utilization at different CO_2_ partial pressure. a) Schematic illustration of CO_2_ utilization in the MEA. b) CO_2_ conversion by reduction or neutralization. c) The predicted local pH values nearby cathodic catalyst surface at variable *j*_total_ when no CO_2_ neutralization happens and d) the predicted local pH values at different *P*_CO2_ conditions based on experiment data of CO_2_ neutralization amount. Mass transfer simulation of e) local [CO_2_] and local [OH^−^], f) [CO_2_]/[OH^−^] ratio and CO_2_R/HER ratio in the MEA‐based model.

The CO_2_ utilization pathway during electrolysis is illustrated in Figure 3a thus providing a roadmap for analysis. The total CO_2_ consumption can be divided into CO_2_ reduction (with the generation of CO and HCOO^−^) and CO_2_ neutralization (forming CO_3_^2−^ and HCO_3_^−^). Among these species, anions (HCOO^−^, CO_3_^2−^ and HCO_3_^−^) will crossover the AEM under an electric field towards the positively charged anode and can potentially be further reacted.^[12]^ HCOO^−^ can be oxidized to CO_2_ at the anode due to it being thermodynamically favored to oxidize before the oxygen evolution reaction (by >1.3 V @ pH=8).^[1a]^ With regards to HCO_3_^−^ and CO_3_^2−^, they can react with protons homogeneously and release CO_2_ at the anode. Using gas chromatography, the amount of CO formation at the cathode and the amount of CO_2_ released at the anode can be calculated. Our previous studies show that formate crossover and oxidation at the anode in the MEA‐based cell limits the total measurable FE at the cathode to <100 %, and we again see that in this work (Figure S3).^[6]^ To quantify the CO_2_ reduction amount to formate, we assumed that the missing charge in FE calculations at the cathode belongs to the formate. Accordingly, the quantitative results relating to CO_2_ utilization at different CO_2_ feed modes are shown in Figure 3b.

The increase of *P*_CO2_ and *j*_total_ results in both a higher CO_2_ consumption amount for reduction and neutralization. However, there is no scaling relation between CO_2_ reduction or neutralization amount with CO_2_ concentration, indicating that the chemical balance in the micro‐environment is changed by the *P*_CO2_. Moreover, the CO_2_ neutralization degree normalized towards the total CO_2_ feed is displayed in Figure S5. It shows that a higher ratio of CO_2_ would participate in the neutralization process when the *P*_CO2_ drops, worsening the [CO_2_]^SA^.

Furthermore, the local pH near the catalyst surface can be estimated by utilizing the quantitative CO_2_ neutralization amounts from the experiment (Figure 3b). Both the CO_2_‐to‐CO half‐reaction and HER produce 1 mol of OH^−^ per 1 mol of e^−^ during the electrolysis process. The CO_2_‐to‐formate (with the OH^−^/e^−^=0.5) is not considered since an unsubstantial amount of the Faradaic efficiency goes towards this reaction. Applying the chronopotentiometry (CP) method allows for the OH^−^ formation rate (mol s^−1^ cm^−2^) to be calculated to predict the local pH. We assumed a fully saturated GDE and uniform catalytic activity throughout the 50 μm catalyst layer.^[9a]^ Figure 3c presents the predicted local pH values at various *j*_total_ in the absence of any CO_2_ neutralization. In general, a larger *j*_total_ allow for a higher alkaline condition at the cathode surface due to the increase of OH^−^ flux, e.g., local pH=13.3 at 100 mA cm^−2^.^[4a]^ By considering the buffer function of CO_2_ neutralization (CO_2_+2OH^−^→CO_3_^2−^+H_2_O), the local pH values at different *P*_CO2_ conditions can be further estimated (Figure 3d). It shows a relatively lower local pH value can be achieved at higher *P*_CO2_ conditions because higher concentrations of CO_2_ are available to participate in the neutralization process, i.e., pH=12.9±0.1 at *P*_CO2_=100 % and pH=13.1±0.1 at *P*_CO2_=25 %. However, the aforementioned simple model ignores the mass‐transfer influences of catalyst layer structure, membrane, *etc*., and thus it can only be used for a qualitative comparison. Nevertheless, the above analysis demonstrates that the CO_2_ neutralization reaction provides an unwanted feedback effect that reduces [CO_2_]^SA^.

Mass‐transport modeling is further employed to verify the above assumptions. A one‐dimensional MEA‐based model (Figure S6) containing a gas diffusion electrode, silver catalyst layer (CL), AEM, and anolyte was developed by a COMOSL multi‐physics field simulation. Dissolved CO_2_ is considered as the reactant in the modelling based on previous works.[^8b^, ^13^] The local HCO_3_^−^, CO_3_^2−^, OH^−^, and CO_2_ concentrations were based on the average value of CL. Figure S7 presents the local HCO_3_^−^ and CO_3_^2−^ concentrations at different operating conditions (*P*_CO2_ and *j*_total_). Their concentrations have a baseline value of ≈100 mM according to the anolyte (100 mM KHCO_3_). It shows the [HCO_3_^−^+CO_3_^2−^] increases with both the *P*_CO2_ and current densities, agreeing with the experimental results (Figure 3b). Moreover, the precise CO_3_^2−^/HCO_3_^−^ ratios (calculated from simulations) at different conditions are provided. The CO_3_^2−^ are the main carrier ions during the electrolysis and this tendency stands out at larger current densities. This result matches with our previous experiments.^[6]^ Figure 3e further demonstrates the calculated local OH^−^ and CO_2_ concentration. At 100 mA cm^−2^, all *P*_CO2_ conditions show a similar local [OH^−^]; while a 2.6 times enhancement of local [OH^−^] is demonstrated when *P*_CO2_ is changed from 100 % to 25 % at 300 mA cm^−2^. More interestingly, increasing the current density leads to a change of local [CO_2_] based on partial pressure, which is expected to be 4 times between *P*_CO2_ of 100 % and 25 %. In fact, a 5‐times local [CO_2_] difference is shown at 300 mA cm^−2^, indicating the additional impact related to CO_2_ neutralization affects the high current electrolysis and lowers the [CO_2_]^SA^. Moreover, the local [CO_2_]/[OH^−^] ratios and CO_2_R/HER ratios are provided in Figure 3f, where a positive correlation is shown between them. We proposed that the local [CO_2_]/[OH^−^] ratio is a good descriptor to reflect the [CO_2_]^SA^, which substantially affects the CO_2_R activity. Comparing with the values of local [CO_2_] or local [OH^−^] alone, this descriptor has the advantage to show the combined effect of the micro‐environment, and especially amplifies the dynamic evolution of [CO_2_] and [OH^−^] at variable operation conditions. Thus, it is of great significance to maintain the high [CO_2_]^SA^ by separately regulating the local [CO_2_] and local [OH^−^] for overcoming the internal limitation of CO_2_ neutralization for large current density electrolysis.

**Figure 4:**
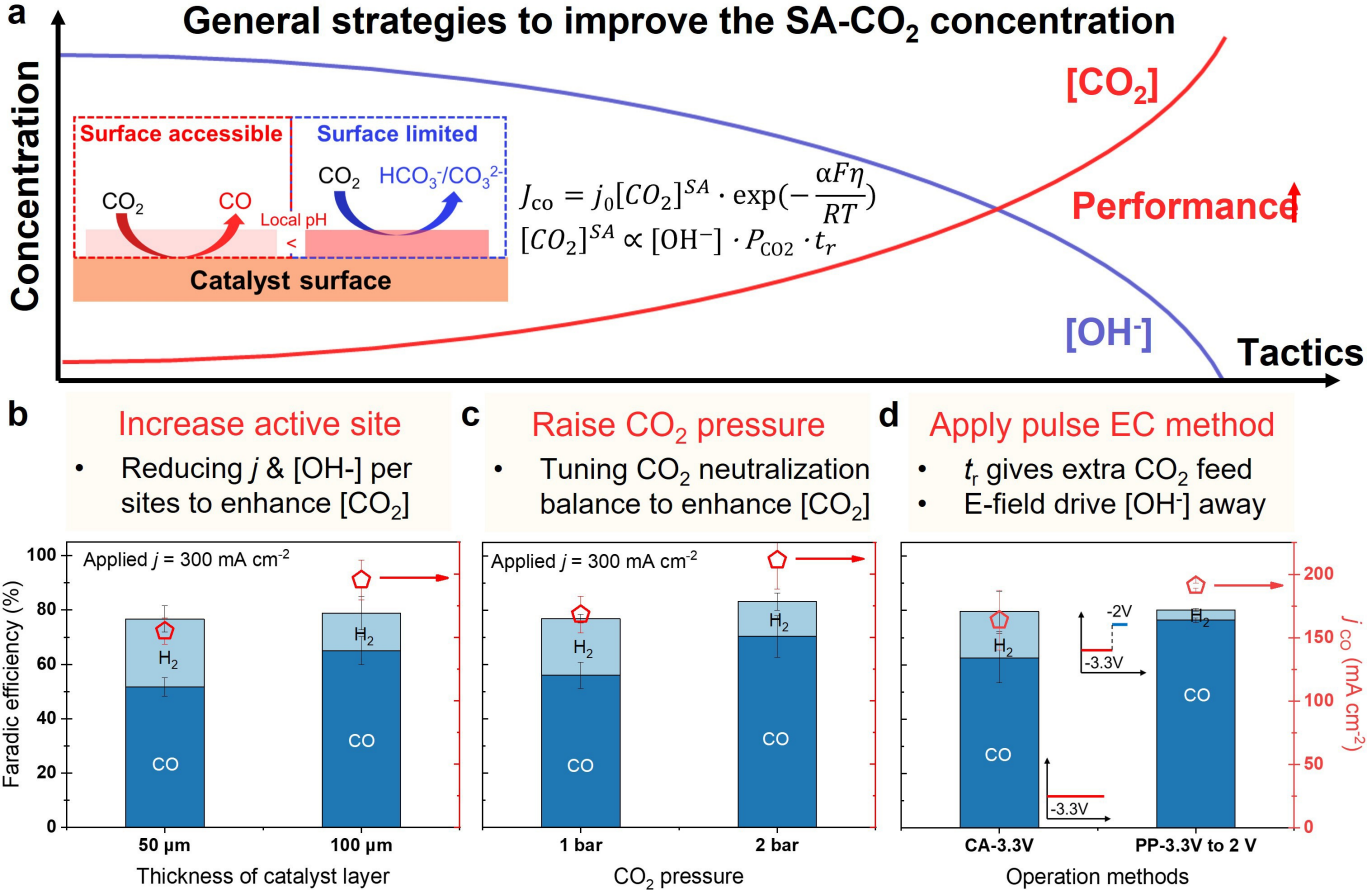
General strategies to enrich the [CO_2_]^SA^. a) Relationship between local [CO_2_], local [OH^−^] and CO_2_R performance. Experimental results of b) increasing catalyst layer thickness, c) elevating CO_2_ pressure (tested in a cell enabling pressurization), and d) applying pulse electrochemical method to enhance the [CO_2_]^SA^.

Mass‐transport modeling has shown that increasing local [CO_2_] will drop the local [OH^−^] and thus improve the [CO_2_]^SA^ on the catalyst for enhancing the *J*_CO_, as displayed in Figure 4a. In principle, the [CO_2_]^SA^ is closely related to the in situ generated [OH^−^] per active site and CO_2_ mass transfer effects (*P*_CO2_, *t*_r_) during the electrolysis period. In cases where pulsed electrolysis is used, *t*_r_ is specific to the given time in the absence of CO_2_R, thus allowing the CO_2_ concentration to recover. Accordingly, we utilized three general strategies to enrich [CO_2_]^SA^ at the catalyst interface: (1) increasing catalyst layer thickness, (2) elevating operating pressure, and (3) applying a pulsed electrochemical technique.

Currently, the main way to compare electrolysis performance under different experimental conditions is using a 2‐dimensional electrode area for denoting current density. Although this way is simple and explicit, it neglects the significant function of catalyst structure and loading on the electrolyzer's performance.^[14]^ For example, by maintaining the catalyst layer volumetric density but increasing its thickness, the cell performance would be substantially modified. This could be enhanced by an increase in the total number of active sites or diminished by lengthening of mass transfer pathway for ions to reach the anode and CO_2_ to penetrate the partially hydrated catalyst layer. Figure 4b shows the selectivity improvement of CO (from 52 %±3 % to 65 %±5 %) with an increase in *j*_CO_ by ca. 40 mA cm^−2^ when the thickness of the catalyst layer was changed from 50 to 100 μm. The 100 μm sample shows a 2‐times larger double‐layer capacitance than the 50 μm sample (see Figure S8 for details), indicating a 2‐times increase in the number of active sites. In this case, the increase of catalyst layer thickness reduces the amount of charge that flows through per active site, thus reducing the in situ generated OH^−^ per active site (local pH) helping to maintain a high [CO_2_]^SA^. The estimated local pH by utilizing the quantitative CO_2_ neutralization amounts also shows the reduction from 13.5±0.2 to 13.2±0.1, verifying the assumption. Moreover, the mass‐transfer modelling result also verifies this assumption, as shown in Figure S9. It shows that the local [CO_2_] raises from 25.7 to 29.3 mM when the catalyst layer thickness increases from 50 μm to 100 μm. However, an increase of catalyst layer thickness will also impede the CO_2_ and H_2_O mass transport, therefore there is an optimal range depending on the porosity and structure of the catalyst distribution within the gas diffusion layer.

The partial pressure experiments demonstrate the degree to which a decrease of *P*_CO2_ will increase the local [OH^−^] and concomitantly decrease the local [CO_2_]. Accordingly, an opposite trend may appear if the CO_2_ total pressure is increased. We show a simple example of this in Figure 4c by increasing the pressure from 1 to 2 bar. As expected, *j*_CO_ is enhanced from 168±14 mA cm^−2^ to 211±23 mA cm^−2^. The increased pressure not only increases the reactant gas density for high local [CO_2_], but also promotes the CO_2_ consumption amount for neutralization which reduces the predicted local pH (from 13.6±0.1 to 13.4±0.1), as shown in Figure S10. These features result in a high [CO_2_]^SA^ allowing for better CO_2_R performance. This demonstration reveals the immense potential of high‐pressure (>10 bar) CO_2_ electrolysis.[^3a^, ^15^] Operating at higher pressures does increase capital costs, so other approaches to increase local [CO_2_] would also be helpful. Thus, another general strategy based on a pulsed electrochemical (EC) method was developed.

Contrary to previous research that utilized the pulsed EC method to regulate the micro‐structure and chemical state of the electrocatalyst (e.g., pulsed anodic of the oxidation potential of the metal catalyst),^[16]^ we attempt to regulate the micro‐environment near the catalyst via [CO_2_] self‐recovery in the MEA‐based electrolyzer. The pulsed method denoted below was operated within the metallic regime of Ag, but at a potential that was either just reductive enough to react with CO_2_ or slightly anodic of this potential, which eliminated Ag oxidation issues and mitigated double‐layer capacitance build‐up transient effects. As a 2‐electrode device was used, the exact cathodic potentials were hard to gauge.

Two operation methods, (1) pulsed potential (PP) and (2) chronoamperometry (CA), are compared in Figure 4d. CA method utilizes a constant cell voltage of 3.3 V while the PP method utilizes a pulsed time period (*t*_a_=1 s) at 3.3 V for the reaction and a recovery time (*t*_r_=0.5 s) at 2.0 V. During the recovery time, the current of cell equals to zero, indicating no Faradaic reaction. To evaluate the FE of CO and H_2_, the concentrations of gas products achieved from the GC data were compensated by the deadtime since the gas flow maintains during the whole pulse period, which reflects the average results including the period where there was no reaction. Remarkably, the PP method allows *j*_CO_ to increase from 164±24 mA cm^−2^ to 191±5 mA cm^−2^ and the FE_H2_ reduces to <4 % as compared to the CA method. Such improvement comes from the recovery period where no CO_2_R occurs but the CO_2_ transfer and neutralization are still continuously happening. This allows [CO_2_]^SA^ to increase during the recovery period while simultaneously permitting anions (e.g., CO_3_^2−^) to leave the local environment as they should be driven away from the negatively charged cathode since there still is an electric field (2 V) between the cathode and anode.

**Figure 5:**
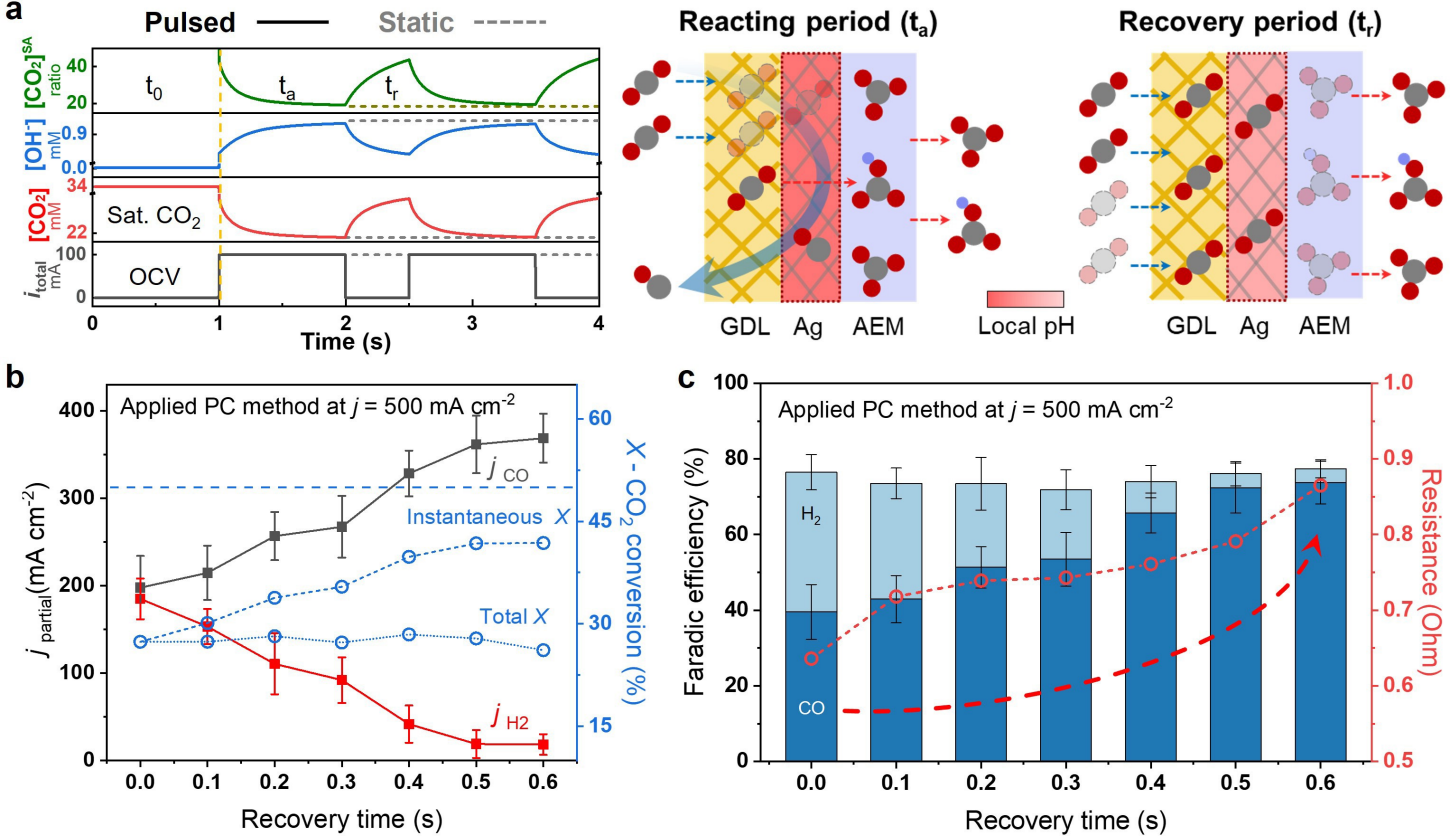
Pulsed‐current (PC) method to optimize the [CO_2_]^SA^. a) Modelling results and schematic illustration of the local [CO_2_], local [OH^−^] and [CO_2_]^SA^ change at reacting or recovery periods during PC method. b) Partial current density of CO, H_2_ and CO_2_ conversion rate (blue straight dot line is *X* of 50 %), c) Faradaic efficiency and cell resistance at different recovery times when applying PC method at 500 mA cm^−2^ with catalyst layer thickness of 100 μm and CO_2_ flow rates of 10 ml min^−1^ cm^−2^.

Literature has demonstrated that a pulsed potential method has shown great promise to regulate the micro‐environment.^[17]^ In addition to pulsed potential, the pulsed current (PC) method may provide more value when the mass transfer is the dominating issue being investigated. The PC method enables one to overcome the mass transfer limitations of the standard chronopotentiometry (CP) method, where we earlier demonstrated insufficient [CO_2_]^SA^. Figure 5a demonstrates the modelling results and schematic illustration of the local [CO_2_] and local [OH^−^] change at reacting or recovery periods during the PC method. To simulate the PC method, a pulse period containing a reacting period (*t*_a_) of 1 s at an *i*_total_=100 mA cm^−2^ and a recovery period (*t*_r_) of 0.5 s at open circuit conditions was repeatedly applied. This shows that the local CO_2_ is saturated and local [OH^−^] is low when no reaction happens (*t*_0_). When applying pulse electrolysis, during the *t*_a_ the local [CO_2_] would gradually drop to a lower degree until reaching steady‐state, and then during the *t*_r_ (if the time was long enough) it would return to near the initial saturated level; meanwhile the local [OH^−^] would vary the opposite way. According to the local CO_2_ recovery and local [OH^−^] mitigation, the PC method allows for a higher [CO_2_]^SA^ during the *t*_a_ compared to the static CP method. Importantly, the *t*_a_ and *t*_r_ values can largely regulate the average [CO_2_]^SA^ during the *t*_a_ period. Experimentally, we operated a *j*_total_ of 500 mA cm^−2^ and optimized the *t*_r_ by fixing the *t*_a_=1 s (Figure 5b, c). The total or instantaneous CO_2_ conversion rate (*X*) is calculated based on the CO_2_ consumption for the CO_2_R divided by the CO_2_ supply during the whole period (*t*_r_+*t*_a_) or only the *t*_a_ period, respectively. As expected, the *j*_CO_, instantaneous *X* and FE_CO_ increase with the *t*_r_. An optimized high *j*_CO_ of 368±28 mA cm^−2^ with FE_CO_ of 74±6 % and instantaneous *X* of 42 % ±4 % can be achieved when *t*_r_≥0.5 s, which is 1.8 times higher than that of the CP method. Further increasing the duration of *t*_r_ shows no increase of the CO_2_R performance implying that the local [CO_2_] is almost recovered when *t*_r_≥0.5 s. It is noted that the total *X* stays at 27±2 % for all *t*_r_ conditions. Actually, the total charge passing the electrode as well as the total in situ formed OH^−^ amount should decrease if they are normalized to the whole period with lengthening *t*_r_. In this case, the maintained total *X* verifies the continuous process of CO_2_ neutralization during *t*_r_ period, which lowers the local [OH^−^] and raises local [CO_2_], resulting in the boost of *j*_CO_ during the *t*_a_ period. Moreover, the cell resistances in Figure 5c increase with the *t*_r_ indicating a higher CO_3_^2−^/OH^−^ ratio in the membrane due to the lower mobility of CO_3_^2−^ than OH^−^ and also a higher CO_2_ neutralization degree during the whole period.^[10a]^

Furthermore, the long‐term stability comparison of the pulse‐current (CP) method with *t*_r_=0.5 s and the normal chronopotentiometry (CP) method at *j*_total_=500 mA cm^−2^ is shown in Figure S11. The *j*_CO_ was calculated based on the reacting period of the two methods. During the first 13 h, the PC method shows much better stability in comparison to the CP method. It still shows a *j*_CO_ of ≈350 mA cm^−2^ with FE_CO_ of ≈70 % at 13 h, which is 2.6 times higher than that of CP method (i.e., a *j*_CO_ of ≈135 mA cm^−2^ with FE_CO_ of ≈27 %). If considering the whole pulse period, the normalized *j*_CO_ is ≈233 mA cm^−2^, which is still 1.7 times higher than that of the CP method. Moreover, after 25 h of CO_2_ electrolysis, the PC method can maintain a total CO_2_ conversion rate higher than 22 % (i.e., an instantaneous conversion rate of 33 %) in contrast to the CP method of only 14 %. The above results illustrate the bright perspective of the pulse EC method to apply for commercially viable CO_2_ electrolysis designs.

Single‐pass CO_2_ conversion (SPC) as a factor to reflect the CO_2_ utilization has attracted great attention recently.^[18]^ In our work, we mainly discussed the internal limitations of CO_2_ neutralization and provide three strategies to enrich [CO_2_]^SA^. These strategies have one general feature: they decrease the [OH^−^] as well as improve the [CO_2_]^SA^ by utilizing CO_2_ neutralization. Therefore, they are not suitable for pursuing high SPC. On the other hand, some advanced cell configurations regarding the electrolyte and membrane designs have been reported to increase the SPC of CO_2_. Their design principle is to create an acidic environment (e.g., acidic electrolytes) or a flow of protons by utilizing a cation‐exchange membrane (CEM) to either directly suppress the CO_2_ neutralization or in situ release the consumed CO_2_.^[18]^ Accordingly, a high SPC of >75 % can be achieved in those configurations, however, they struggle with large ohmic loss in their setups.

Another important research area for CO_2_ reduction is to precisely measure the local pH and CO_2_ concentration near the catalyst surface (within micro‐ or nano‐meters).^[19]^ Significant progress have been made. For example, Monteiro et al. reported a time‐resolved local pH measurements during CO_2_ reduction by using scanning electrochemical microscopy.^[20]^ They detected a plateau region of pH nearby the catalyst surface during CO_2_R due to the formation of HCO_3_^−^ buffering the reaction interface. This kind of experimental results are useful to estimate the real local pH values across the diffusion layer, thus further helping to develop the model and improve the modelling parameters. However, until recently, it is still a challenge to in situ characterize the local pH/CO_2_ concentration in MEA‐based setups due to the complex interfacial structure. Thus, mass‐transfer modelling and calculations play an important role in supporting the experimental results.^[21]^ We advocate to further develop operando spectroscopy and microscopy for the MEA‐based cell and extract knowledge from these techniques to optimize the theoretical models, achieving a better understanding on the CO_2_ electrolysis.

## Conclusion

In this work, we systematically explored the change of CO_2_ neutralization in terms of local [CO_2_] and local [OH^−^] near the catalyst layer by regulating the CO_2_ feed mode (e.g., flow rate, partial pressure, *etc*.) and applied current densities in a zero‐gap AEM‐based electrolyzer. The quantitative analyses and theoretical calculation results together reveal that the [CO_2_]^SA^ (i.e., local [CO_2_]/[OH^−^]), which has the advantage of amplifying the dynamic evolution of local [CO_2_] and local [OH^−^] in the micro‐environment at different operation conditions, is a good descriptor for CO_2_R performance. A higher [CO_2_]^SA^ is favorable for electrochemical CO_2_ to CO conversion.

In principle, *P*_CO2_ and *j*_total_ help set the initial local [CO_2_] and local [OH^−^], respectively; whereas once electrolysis proceeds, they would chemically balance with each other until reaching steady state. Increasing the initial *P*_CO2_ and decreasing the *j*_total_ will both render a lower ratio of CO_2_ to participate in the neutralization process, leading to a high local [CO_2_] and a low local [OH^−^], i.e., improving the [CO_2_]^SA^. Moreover, CO_2_ neutralization is a dynamic process requiring some period of time to reach equilibrium. This phenomenon creates a possibility to utilize the dynamic evolution of the local [CO_2_] and [OH^−^] to keep the [CO_2_]^SA^ at a high level, such that one may overcome the internal limitations of CO_2_ neutralization. Based on these understandings, three general strategies were developed to enrich [CO_2_]^SA^ for high current electrolysis: (1) increasing the catalyst layer thickness to reduce the *j*_total_ per active sites, (2) elevating CO_2_ pressure (e.g., *P*_CO2_>1 bar), and (3) employing pulse electrochemical method to keep the [CO_2_]^SA^ at a high level. Consequently, a commercial silver baseline catalyst achieved a *j*_CO_ of 368±28 mA cm^−2^ with FE_CO_=74±6 % via an optimized pulsed‐electrochemical method.

## Conflict of interest

The authors declare no conflict of interest.

1

## Supporting information

As a service to our authors and readers, this journal provides supporting information supplied by the authors. Such materials are peer reviewed and may be re‐organized for online delivery, but are not copy‐edited or typeset. Technical support issues arising from supporting information (other than missing files) should be addressed to the authors.

Supporting Information

## Data Availability

The data that support the findings of this study are available from the corresponding author upon reasonable request.
