# Supplementary material for: Enriching Surface‐Accessible CO2 in the Zero‐Gap Anion‐Exchange‐Membrane‐Based CO2 Electrolyzer
Source: Angew Chem Int Ed Engl. 2022 Dec 13;62(3):e202214383. doi: 10.1002/anie.202214383 (PMC10108229; doi:10.1002/anie.202214383)
Supplement: Supplementary file 1 — Supporting Information [file ANIE-62-0-s001.pdf]

Angewandte Chemie
Eine Zeitschrift der Gesellschaft Deutscher Chemiker

Supporting Information

Enriching Surface-Accessible CO_2 in the Zero-Gap Anion-Exchange-Membrane-Based CO_2 Electrolyzer

Q. Xu, A. Xu, S. Garg, A. B. Moss, I. Chorkendorff, T. Bligaard, B. Seger**

Table of Contents

Section 1. Experimental Procedures

1.1 Zero-gap MEA-based electrolyzer configuration
1.2 Experiment conditions
1.3 Characterization and electrochemical measurements
1.4 Mass-Transport Simulation (contains Table S1 & S2)

Section 2. Supporting Figures

Figure S1. The SEM image and XPS Ag 3d spectrum of silver membrane.
Figure S2. The cell voltage, j_{CO} and faradaic efficiency at different applied current densities and CO_2 flow rates.
Figure S3. The cell voltage, j_{CO} and faradaic efficiency at different applied current densities and CO_2 partial pressure.
Figure S4. The predicted CO_2 partial pressures at cathodic outlet ($P_{CO2\text{-}outlet}$) for different CO_2 flow rates.
Figure S5. Comparison of the CO_2 neutralization degree at different CO_2 partial pressure.
Figure S6. The configuration of one-dimensional MEA-based model for mass-transport simulation.
Figure S7. The local [CO_3^{2-}+HCO_3^-] concentration and CO_3^{2-}/HCO_3^- ratio in the MEA-based model.
Figure S8. Double-layer capacity of the Ag-M samples with different thickness.
Figure S9. Modelling results of the local [CO_2] for samples with catalyst layer thickness of 50 μm or 100 μm.
Figure S10. The predicted local pH at CO_2 pressure of 1 bar and 2 bar.
Figure S11. Stability comparison of pulse-current (PC) method and normal chronopotentiometry (CP) method.
Figure S12. The flow diagram of the CO_2 electrolysis setup.

Section 3. Supporting References

Section 4. Author Contributions

Section 1: Experimental Procedures

1.1 Zero-gap MEA-based electrolyzer configuration

Electrochemical CO_2 reduction tests were performed in a custom-made electrolyzer equipped with graphite and titanium serpentine flow fields for the cathode and anode, respectively. The zero-gap MEA was assembled by placing an anion-exchange membrane (Sustainion X37-50-grade RT, Dioxide Materials, 6.25 cm^2) between the anode and cathode electrode. A porous silver membrane with a single layer thickness of 50 μm and an average pore size of 5 μm (Sterlitech Inc., purity 99.97%, porosity of ~55%, 1 cm^2) was used as a cathodic catalyst layer. Toray Paper (TGP-H-060) as a gas-diffusion layer was attached to the silver membrane and together they worked as the cathode. An IrO_2-coated carbon paper electrode (Dioxide Materials, 2.25 cm^2) was used as the anode. During assembly, the MEA was surrounded by fluorinated-ethylene-propylene (FEP) gaskets and the bolts of the electrolyzer were fastened with a torque of 4 N·m.

1.2 Experiment conditions

The cathodic flow field was fed with pure CO_2 gas (AGA, 4.5 N) or mixed high-pure gas with flow rates ranging from 5 to 20 $mL\cdot min^{-1}\cdot cm^{-2}$ by using a mass flow controller. CO_2 partial pressure was regulated by controlling the mass flow rate of CO_2 (as reactant) and Ar (as inert gas) through two mass flow controllers (Vöegtlin red-y smart series and MKS G Series). The outlet cathode gas stream was passed through a gas wash (20 ml pure water) to extract the liquid CO_2RR products. The anodic flow field was fed with 0.1 M $KHCO_3$ anolyte (40 ml, prepared from 99.99% metal basis chemicals, Sigma-Aldrich) by a diaphragm pump (KNF NF1.5TTDCB-4). The anolyte reservoir was bubbled with Ar (20 $mL\cdot min^{-1}$) to carry the gases formed at the anode for gas chromatography (GC) analysis. The flow rate of the cathodic and anodic outlet streams of the electrolyzer was further measured with a volumetric flow meter (GFM Pro, Thermo Scientific). The flow diagram of the electrolysis setup is exhibited in **Figure S12**. Raising CO_2 pressure experiments were performed on an another custom-made electrolyzer with a reinforced tubing connection that allows us to operate at high backpressure conditions. CO_2 pressure was controlled at both cathode and anode sides using two Equilibar LF Series back pressure regulators in PEEK. For both the pilot pressure was controlled by electronic pressure controllers (PCS-DRP70). All gas products were analyzed using the GC (PerkinElmer Clarus 580 or Agilent 6890A) equipped with a thermal conductivity detector and a flame ionization detector.

1.3 Characterization and electrochemical measurements

The surface morphology of porous Ag electrodes was measured by scanning electron microscopy (SEM, FEI Quanta 200) at an accelerating voltage of 15 kV. Surface chemical state analysis was measured by X-ray photoelectron spectroscopy (XPS, Thermo Scientific™ Theta Probe) equipped with a monochromated Al Kα radiation (1486.7 eV).

All electrochemical tests were performed in the two-electrode system by using a potentiostat (Biologic VSP-89). All measured cell voltages were reported without any *iR* correction. Faradaic efficiency (FE) was calculated by equation as $FE_i = (n\cdot F\cdot C_i\cdot V)/I_{total}$, where n is the number of electrons transferred, F is Faraday's constant, C_i is the molar concentration of species i, V is the total volumetric flow rate, and I_{total} is the applied current at chronopotentiometry (CP) mode or the measured total current at potentiostatic mode. CO_2 utilization rate is calculated by dividing total CO_2 reduction product (CO and $HCOO^-$) to the total CO_2 supply. For pulsed electrochemical method, the concentrations of gas products achieved from the GC data required to be compensated by the deadtime since the gas flow maintains during the whole pulse periods. Thus, the as exhibited performance only reflects the reacting period.

1.4 Mass-Transport Simulation

The model curves in the **Fig. 1b** are simulated based on the concentration-dependent Butler-Volmer equation, as shown below:

$$j = j_0[CO_2]^{surf} exp\left\{\frac{(1-\beta)F\eta}{RT}\right\} \quad \text{eq.1}$$

where j or j_0 is the electrode current density or the exchange current density; β is the charge transfer coefficient; F is the Faraday constant of 96485 C/mol; T is the absolute temperature of 298.15 K; R is the universal gas constant of 8.314 J/(K·mol); η is the activation overpotential.

A one-dimensional MEA-based model was developed by the COMOSL multi-physics field simulation. The configuration of model is exhibited in **Figure S6**, which comprises a 190 μm gas diffusion electrode (GDE, porosity of 0.78), a 50 μm Ag membrane cathodic

catalyst layer (CL, porosity of 0.55), and a 50 μm anion-exchange membrane (AEM). The electrochemical CO_2-to-CO conversion, HER and CO_2 neutralization reactions (CO_2-HCO_3^--CO_3^{2-} equilibrium) are involved in the model for estimate the local CO_2 and OH^- concentrations. The transport of gas (CO_2, CO, H_2) and liquid electrolyte are simulated using a mixture diffusion model (eq.2)

$$\frac{dc_i}{dt} = -\rho_g D_i^{eff} \nabla\omega_i - \rho_g D_i^{eff} \omega_i \frac{\nabla M_n}{M_n} \quad \text{eq.2}$$

where ω_i, ρ_g and M_n are the mass fraction of species i, ideal gaseous mixture density and average molar mass. The boundary conditions are listed in the **Table S1**.

We used Maxwell-Stefan diffusivity to represent the effective diffusion coefficient D_i^{eff},

$$D_i^{eff} = \frac{1-\omega_i}{\sum_{n\neq i}\frac{y_n}{D_{in}}} \quad \text{eq.3}$$

where y_n is the molar fraction of species n. D_{in} can be found in parameters table (**Table S2**).

The transport of species in liquid electrolyte are simulated using Nernst-Planck equation (eq.4) and the potential field is solved by the Poisson equation (eq.5),

$$\frac{dc_i}{dt} = D_i \frac{d^2c_i}{dx^2} + \frac{D_i z_i e}{k_B T} C_i \frac{d^2\varphi}{dx^2} + \frac{D_i z_i e}{k_B T} \frac{dc_i}{dx} \frac{d\varphi}{dx} \quad \text{eq.4}$$

$$-\varepsilon\varepsilon_0 \frac{d^2\varphi}{dx^2} = dx * \sum_i z_i e c_i \quad \text{eq.5}$$

where c_i, D_i, z_i are the concentration, diffusion coefficient and charge of a specific substance; x is the position relative to the electrode and t is time; φ is the electrolyte potential, ε and ε_0 are the permittivity of vacuum and water.

We included the following equilibrium reactions in liquid electrolyte,

$$CO_2 + OH^- \underset{k^{1-}}{\overset{k^{1+}}{\rightleftharpoons}} HCO_3^- \quad \text{eq.6}$$

$$HCO_3^- + OH^- \underset{k^{2-}}{\overset{k^{2+}}{\rightleftharpoons}} CO_3^{2-} \quad \text{eq.7}$$

Table S1. Boundary conditions of mass-transport simulation

$x = 0$	$\omega_i = \omega_{i,inlet}$ (depending on CO_2 partial pressure)
$x = \delta_{GDE} + \delta_{Ag} * (1 - s)$	$f_i = K_{GL,i}(c_i^* - c_i)$ (gas-to-liquid mass transfer)
$x = \delta_{GDE} + \delta_{Ag}$	$c_i^{Ag} = c_i^{Mem}$, $\varphi = \varphi^{app}$
$x = \delta_{GDE} + \delta_{Ag} + \delta_{Mem}$	$c_i^{Mem} = c_i^{BL}$, $\varphi^{Mem} = \varphi^{BL}$
$x = \delta_{GDE} + \delta_{Ag} + \delta_{Mem} + \delta_{BL}$	$c_i^{BL} = c_i^{bulk}$, $\varphi = 0\ V$

Table S2. Parameters table of mass-transport simulation.

Parameters	Value or definition	Unit	ref
$D_{H2\text{-}CO}$	0.743	$cm^2\ s^{-1}$	[1]
$D_{H2\text{-}CO2}$	0.646	$cm^2\ s^{-1}$	[1]
$D_{CO\text{-}CO2}$	0.152	$cm^2\ s^{-1}$	[1]
D_{K^+}	1.96e-5	$cm^2\ s^{-1}$	[2]
$D_{HCO_3^-}$	1.10e-9	$cm^2\ s^{-1}$	[2]
$D_{CO_3^{2-}}$	0.80e-9	$cm^2\ s^{-1}$	[2]
D_{OH^-}	4.93e-9	$cm^2\ s^{-1}$	[2]
$D_{CO_2(aq)}$	1.91e-9	$cm^2\ s^{-1}$	[2]
$D_{H_2(aq)}$	5.11e-9	$cm^2\ s^{-1}$	[2]

$D_{CO(aq)}$	2.03e-9	$cm^2\ s^{-1}$	[2]
$K_{GL,i}$	$D_i/\delta_{liquid\ film}$	$cm\ s^{-1}$	[1]
$\delta_{liquidfilm}$	10	nm	[3]
c^*_{CO2}	34	mmol/L	[3]
c^*_{CO}	1	mmol/L	[3]
c^*_{H2}	1	mmol/L	[3]
δ_{GDE}	190	µm	measured
δ_{Ag}	50	µm	measured
s	0.64	µm	[4]
δ_{Mem}	50	µm	measured
δ_{BL}	100	µm	assumed
k^{1+}	2.23e3	$(M\ s)^{-1}$	[5]
k^{1-}	9.71e-5	s^{-1}	[5]
k^{2+}	6.50e9	$(M\ s)^{-1}$	[5]
k^{2-}	1.34e6	s^{-1}	[5]
β_{CO2}	0.5		assumed
β_{H2}	0.5		assumed
$j_{0,H2}$	1.16e-6	$mA\ cm^{-2}$	[3]
$j_{0,CO2}$	4.71e-4	$mA\ cm^{-2}$	[3]

Section 2: Supporting Figures

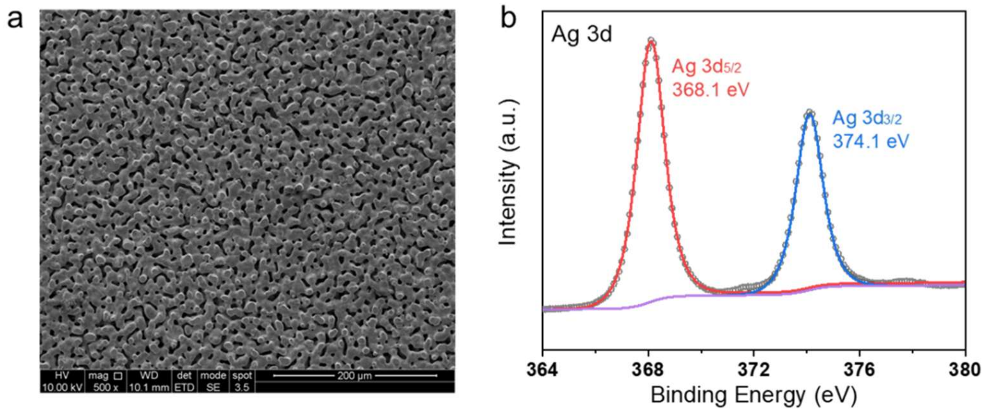

Figure S1. a) The SEM image, b) XPS Ag 3d spectrum of silver membrane.

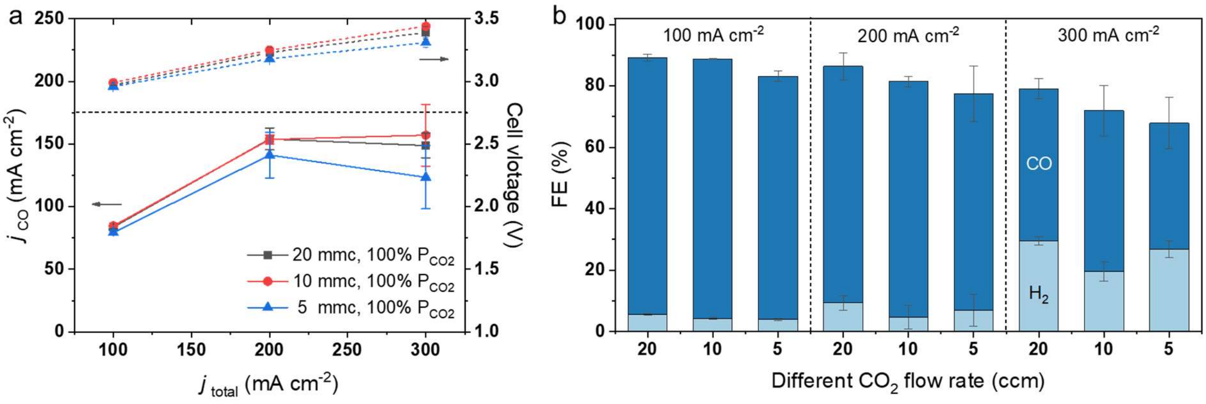

Figure S2. a) The cell voltage and j_{CO}, b) faradaic efficiency at different applied current densities and CO_2 flow rates. The unit of mL·min^{-1}·cm^{-2} (mmc) represents the CO_2 flow volume per minute per active electrode area.

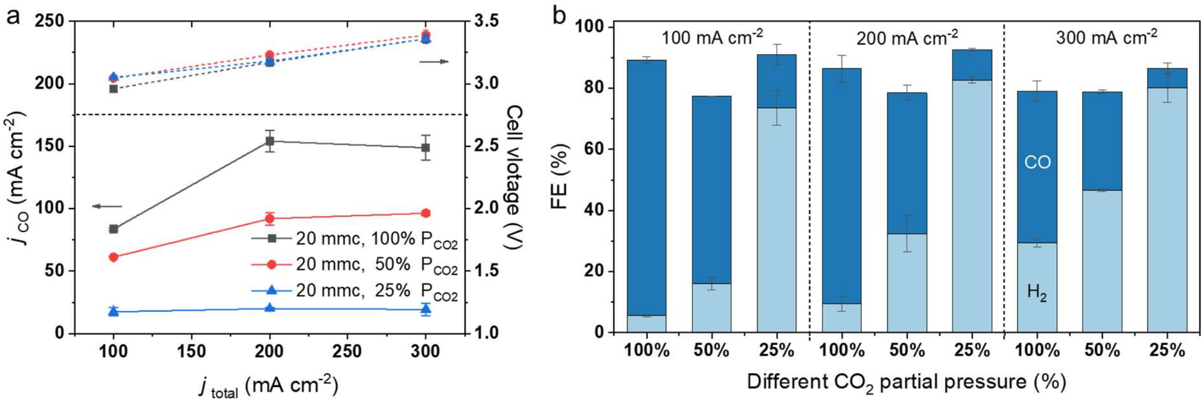

Figure S3. a) The cell voltage and j_{CO}, b) faradaic efficiency at different applied current densities and CO_2 partial pressure. The unit of mL·min^{-1}·cm^{-2} (mmc) represents the mixed gas (CO_2+Ar) flow volume per minute per active electrode area.

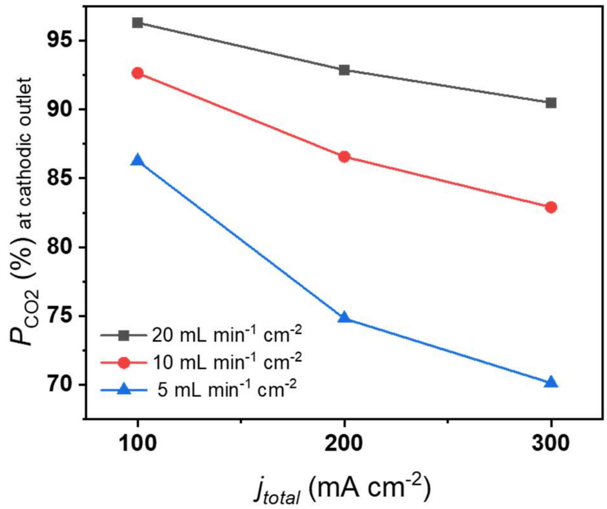

Figure S4. The predicted CO_2 partial pressures at cathodic outlet ($P_{CO2\text{-outlet}}$) for different CO_2 flow rates (mL min^{-1} cm^{-2}). The CO_2 partial pressures at the inlet for all conditions are 100%. The CO_2 partial pressure at the outlet was calculated by equation: $P_{CO2\text{-outlet}}$ = CO_2 outlet flow / (CO_2 + H_2 + CO outlet flow), where the CO_2 outlet flow considered the CO_2-to-CO conversion and CO_2 neutralization, H_2 and CO outlet flow was calculated based on their FE values from the experiment results. The CO_2 consumption amount for neutralization is fixed as 10% of total CO_2 feed.

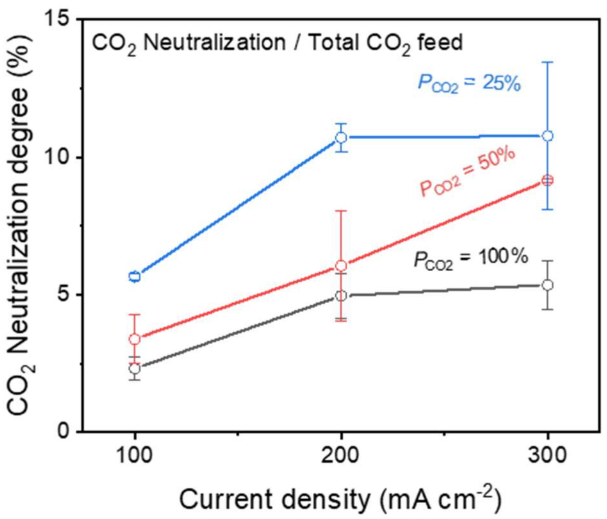

Figure S5. Comparison of the CO_2 neutralization degree at different CO_2 partial pressure.

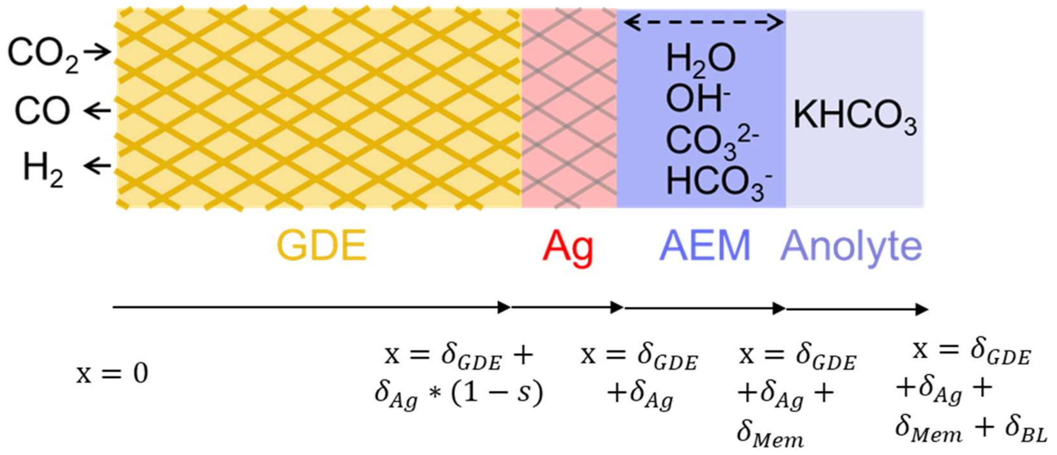

Figure S6. The configuration of one-dimensional MEA-based model for mass-transport simulation.

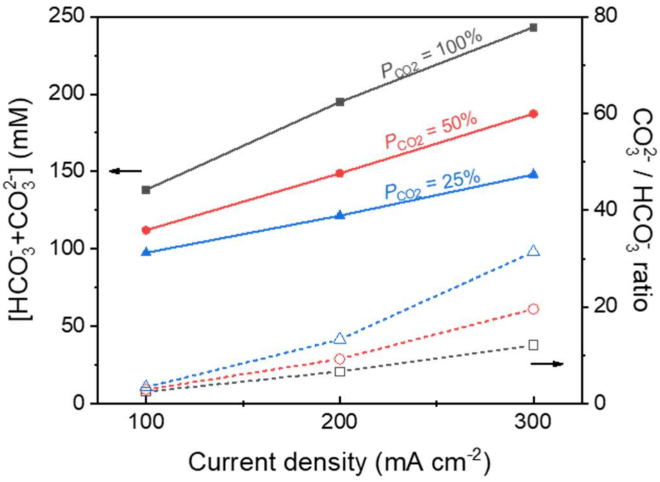

Figure S7. The local $[CO_3^{2-}+HCO_3^-]$ concentration and CO_3^{2-}/HCO_3^- ratio in the MEA-based model.

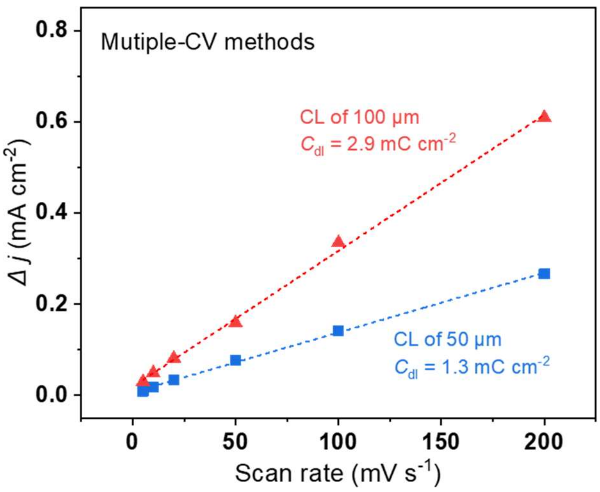

Figure S8. Double-layer capacity of the Ag-M samples with different thickness (CL of 50 or 100 μm) measured by multiple-CV method.

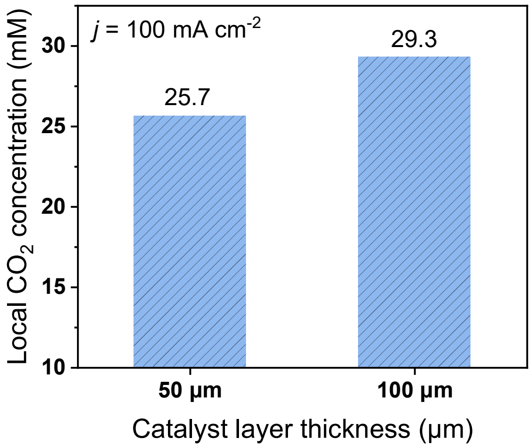

Figure S9. Mass transfer modelling results of the local CO_2 concentration for samples with catalyst layer thickness of 50 μm or 100 μm at 100 mA cm^{-2}.

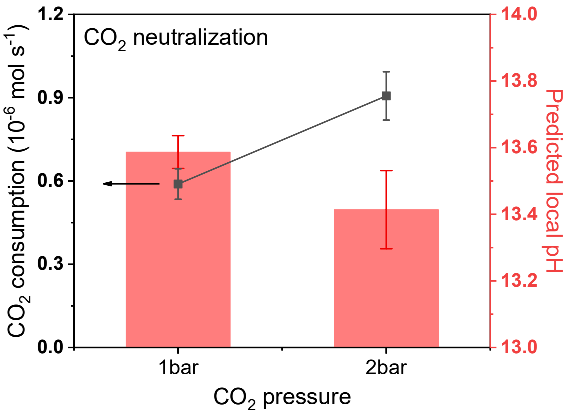

Figure S10. The measured CO_2 consumption amount for neutralization and the predicted local pH at CO_2 pressure of 1 bar and 2 bar.

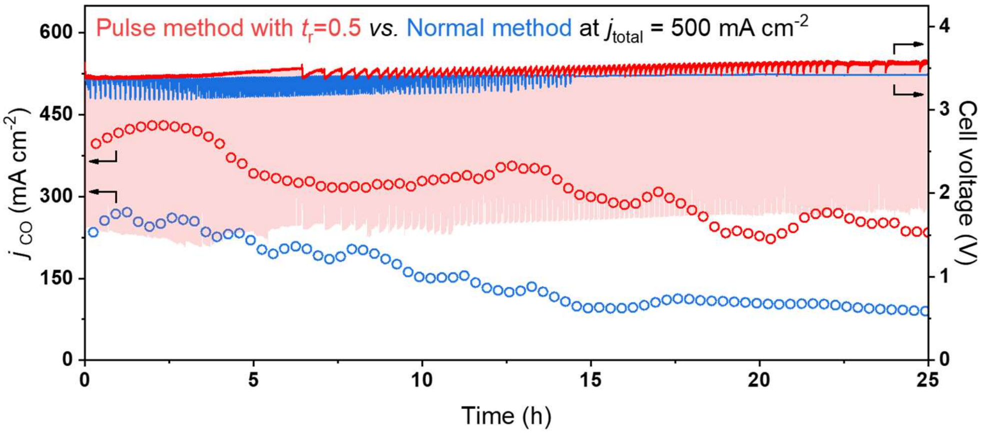

Figure S11. Stability comparison of pulse-current (PC) method with t_r=0.5 s and normal method of chronopotentiometry (CP) at j_{total} = 500 mA cm^{-2} with catalyst layer thickness of 100 μm and CO_2 flow rates of 10 ml min^{-1} cm^{-2}.

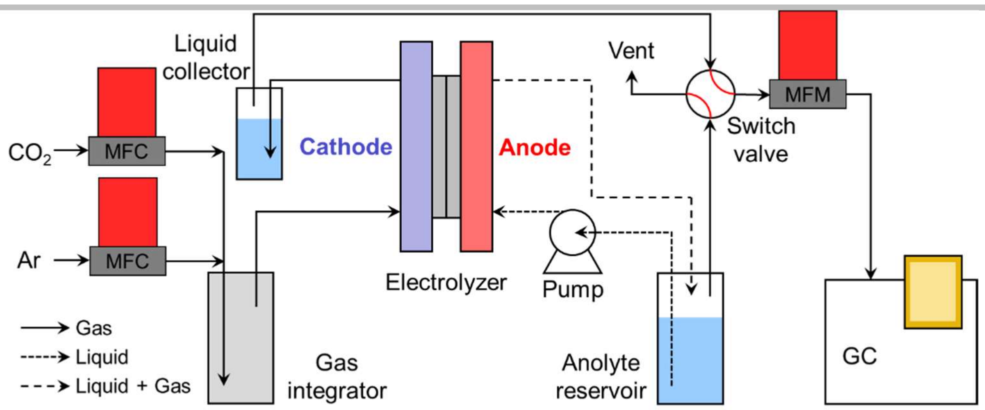

Figure S12. The flow diagram of the CO_2 electrolysis setup.

Section 3: Supporting References

[1] E. L. Cussler, E. L. Cussler, *Diffusion: mass transfer in fluid systems*, Cambridge university press, **2009**.
[2] J. Newman, K. E. Thomas-Alyea, *Electrochemical systems*, John Wiley & Sons, **2012**.
[3] L.-C. Weng, A. T. Bell, A. Z. Weber, *Physical Chemistry Chemical Physics* **2018**, *20*, 16973-16984.
[4] A. El-Kharouf, T. J. Mason, D. J. Brett, B. G. Pollet, *Journal of Power sources* **2012**, *218*, 393-404.
[5] K. G. Schulz, U. Riebesell, B. Rost, S. Thoms, R. Zeebe, *Marine chemistry* **2006**, *100*, 53-65.

Section 4: Author Contributions

Q. Xu contributed to the experiment design, data analysis and wrote the manuscript. A. Xu built the one-dimensional MEA-based model and performed the mass-transport simulation. Q. Xu, A. Xu and S. Garg contributed to the discussion on data. A. B. Moss helped on the high-pressure MEA-based electrolyzer setup. I. Chorkendorff and Thomas Bligaard guided this work. B. Seger supervised and guided the whole project.
